# Urinary 2,8-dihydroxyadenine crystals in a patient with adenine phosphoribosyltransferase deficiency

**DOI:** 10.1093/qjmed/hcad124

**Published:** 2023-06-07

**Authors:** T Horino, M Ishihara, M Fujieda

**Affiliations:** Department of Endocrinology, Metabolism and Nephrology; Department of Paediatrics, Kochi Medical School, Kohasu, Kochi 783-8505, Japan; Department of Paediatrics, Kochi Medical School, Kohasu, Kochi 783-8505, Japan

Learning point for clinicians Delayed diagnosis of APRT deficiency causes progression to irreversible renal failure. GC/MS-based metabolomics assessment is non-invasive, quick and accurate, and therefore useful for the early diagnosis of APRT deficiency, allowing effective treatment.

##  

A 21-year-old man was admitted to our hospital for a consultation regarding abnormal urine. The patient had no history of urolithiasis. A urinalysis showed characteristic annular crystals with central spicules (Figure 1), without proteinuria or haematuria. Laboratory data revealed serum creatinine and uric acid levels of 0.82 and 6.0 mg/dL, respectively. The urinary metabolome was analysed using gas chromatography-mass spectrometry (GC/MS); results showed elevated levels of urinary 2,8-dihydroxyadenine (DHA; 4·6 SD), 8-hydroxyadenine (4·5 SD) and adenine (4·2 SD). Based on the above findings, adenine phosphoribosyltransferase (APRT) deficiency was diagnosed. Treatment with 40 mg/day topiroxostat, a xanthine oxidoreductase (XOR) inhibitor, resulted in the rapid disappearance of 2,8-DHA crystals from the patient’s urine.

**Figure 1.**
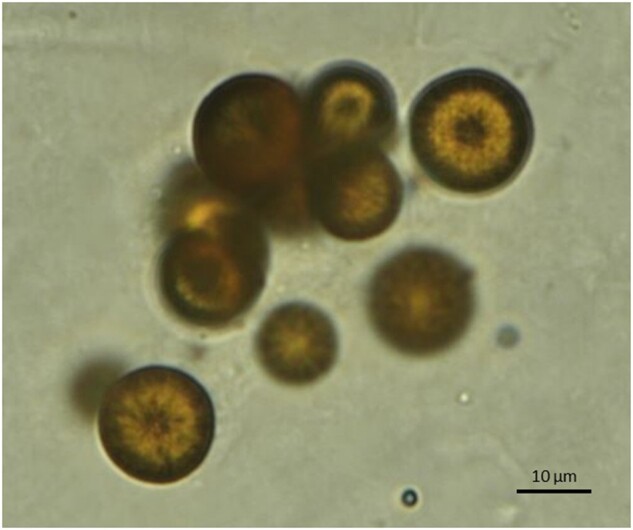
Findings of urine sediment. Light microscopy of urine sediment showing 2,8-DHA crystals which are brownish with a darker outline and spicules radiating from the centre.

APRT deficiency is a rare autosomal recessive disorder of adenine metabolism, with the most common symptom being crystalline nephropathy or recurrent urolithiasis; 2,8-DHA crystals in urine can be observed microscopically.^1^^,^^2^ In patients with APRT deficiency, adenine accumulates and is converted to large amounts of 2,8-DHA by XOR.^1^ A large proportion of patients remain asymptomatic for years, leading to a delayed diagnosis.^1^^,^^2^ Therefore, many patients have already developed irreversible renal failure when treatment is implemented, and approximately 15% have end-stage kidney disease at the time of diagnosis.^1^^,^^2^ Because XOR inhibitors are effective against the progression of 2,8-DHA crystalline nephropathy, early diagnosis and therapeutic intervention with XOR inhibitors are warranted.^1^^,^^2^ The disease is currently under-reported in the majority of the patients who therefore do not receive the benefits of medical therapy.^3^ The reasons are a lack of familiarity with 2,8-DHA stones among clinicians/pathologists, the lack of routine stone analysis and confusion of 2,8-DHA stones with radiolucent uric acid and/or xanthine stones. Genetic testing for mutations known to abolish APRT enzyme activity is a useful tool for diagnosing APRT deficiency, but genetic testing alone is insufficient,^2^ resulting in an inconclusive diagnosis. An additional disadvantage of this method is that, in clinical practice, patients are often reluctant to undergo genetic testing.^4^ Metabolomic evaluation is an omics analysis method that comprehensively analyses hundreds of metabolites.^4^ An advantage of metabolomic evaluation is that it enables quick and easy chemical diagnosis of rare diseases, and allows the acquisition of highly accurate biological information using small specimens obtained non-invasively.^4^ GC/MS-based metabolomic assessment is therefore useful for the early diagnosis of APRT deficiency.

## Patient consent

The patient provided written consent for publication of the images.

## Data Availability

Data are available upon reasonable request by any qualified researchers, who engage in rigorous, independent scientific research, and will be provided following review and approval of a research proposal and Statistical Analysis Plan and execution of a Data Sharing Agreement. All data relevant to this study are included in the article.
